# Removal of leftover feed shapes environmental microbiota and limits houseflies-mediated dispersion of pathogenic bacteria in sow breeding farms

**DOI:** 10.1186/s42523-024-00296-6

**Published:** 2024-03-05

**Authors:** Yunke Li, Yinfeng Chen, Zhaohui Chen, Ying Yang, Zhenlong Wu

**Affiliations:** 1 State Key Laboratory of Animal Nutrition and Feeding, China Agricultural University, Beijing, 100193 China; 2 Beijing Advanced Innovation Center for Food Nutrition and Human Health, China Agricultural University, Beijing, 100193 China; 3 Laboratory of Microbial Resources and Application in Animals, Beijing Jingwa Agricultural Science and Technology Innovation Center, Pinggu Beijing, Beijing, 101206 China

**Keywords:** All-in/all-out system, Housefly, Environmental microbial transmission, Leftover feed, Gestating sow

## Abstract

**Background:**

Intensive swine breeding industry generates a complex environment where several microbial interactions occur and which constitutes a challenge for biosafety. Ad libitum feeding strategies and low levels of management contribute to residual and wasted feed for lactating sows, which provides a source of nutrients and microbial source for houseflies in warm climates. Due to the absence of the all-in/all-out system, the coexistence of sows of two production stages including gestating and lactating sows in the farrowing barn may have potential negative impacts. In this research, we evaluated the effects of lactating sow leftover on the environmental microbiota of the farrowing barn and the contribution of microbial environments to the gestating sow fecal bacterial structure with a 30-day-long treatment of timely removing lactating residual feed.

**Results:**

Houseflies in the farrowing barn mediate the transmission of microorganisms from lactating sow leftover to multiple regions. *Leuconostoc*, *Weissella*, *Lactobacillus* and *Pediococcus* from the leftover which can produce exopolysaccharides, are more capable of environmental transmission than pathogenic microorganisms including *Staphylococcus* and *Streptococcus* and utilize houseflies to achieve spread in environmental regions of the farrowing barn. Leftover removal treatment blocked the microbial transmission chain mediated by houseflies, downregulated the relative abundance of pathogenic bacteria including *Escherichia-Shigella* and *Streptococcus* among houseflies, environmental regions and fecal bacteria of gestating sows in the farrowing barn and effectively attenuate the increment of *Weissella* and *RF39* relative abundance in gestating sow feces due to the presence of lactating sows.

**Conclusions:**

Lactating sow leftover is a non-negligible microbial contributor of environment in farrowing barn whose transmission is mediated by houseflies. A 30-day-long treatment of removing lactating sow residual feed cause significant changes in the microbial structure of multiple environmental regions within the farrowing barn via altering the microbiota carried by houseflies. Meanwhile, lactating sow leftover affect the fecal microbial structure of gestating sows in the same farrowing barn, while removal of lactating sow leftover alleviates the contribution of microbial transmission.

**Supplementary Information:**

The online version contains supplementary material available at 10.1186/s42523-024-00296-6.

## Background

Livestock farms have complex microbiological environments, while the risk of microbial dispersion is being increasingly emphasized [1, 2]. Efficient nutritional formulations [3], feeding and management strategies [4] can reduce production costs and limit the spread of pathogenic microorganisms, including the all-in/all-out (AIAO) system. The AIAO system means there is only one stage of production and health state of livestock animals in each barn. However, the AIAO system has not yet been implemented in some developing country farms due to cost constraints including space and equipment.

Primiparous and multiparous sows are housed in the breeding barn or farrowing barn based on their gestational age. Farms that do not strictly implement an AIAO systems often confront with the situation where lactating sows and gestating sows are housed together in the same farrowing barn. Meanwhile, in order to increase the productivity of sows during gestation, restricted feeding during gestation and ad libitum feeding after farrowing are often adopted [5], which upregulates the complexity of management in mix-feeding farrowing barns and also results in the residual of leftover feed for lactating sows. When the temperature becomes warm, feed residual may become a source of nutrients for arthropods while simultaneously serving as a colony for microorganisms.

Houseflies (*Musca domestica* L.) are common arthropods found on farms whose life expectancy and reproductive capacity are closely associated with their compatible breeding regional choices, including the availability of excrement, decaying organic matter and waste [6], which are conditions available in livestock farms. Meanwhile, warm climates can shorten the egg stage of housefly to 7–10 days [7], causing the proliferation of houseflies. Houseflies can transmit microorganisms by physical contact and have been demonstrated to mediate seasonal transmission of pathogens such as *Shigella* [8] and *Streptococcus* [9] in areas of human activity, as well as *Staphylococcus aureus* [10], multidrug-resistant *Salmonella* [11] and porcine reproductive and respiratory syndrome virus [12] in swine farms. However, the role of houseflies in influencing microbial environment within farms has not been well demonstrated.

Recent researches have demonstrated the impact of environmental factors on the host microbiome [13]. Comparing to outdoor environments, the indoor microbial environments are often overlooked but equally important [14]. Meanwhile, growing evidence shows that the microbial structure of the perinatal maternal digestive tract is fragile [15, 16] and can be modified by several factors including exogenous environmental microorganism colonization [17]. Nowadays, intestinal or fecal microbiota is recognized as a key player for assessing the external environments [18, 19], which makes gestating sow suitable samples for evaluating changes in environmental microbial structure and the impact on maternal microbiota in the mix-feeding barn due to the failure to implement the AIAO system.

## Method

### Animals and experimental design

**Fig. 1:**
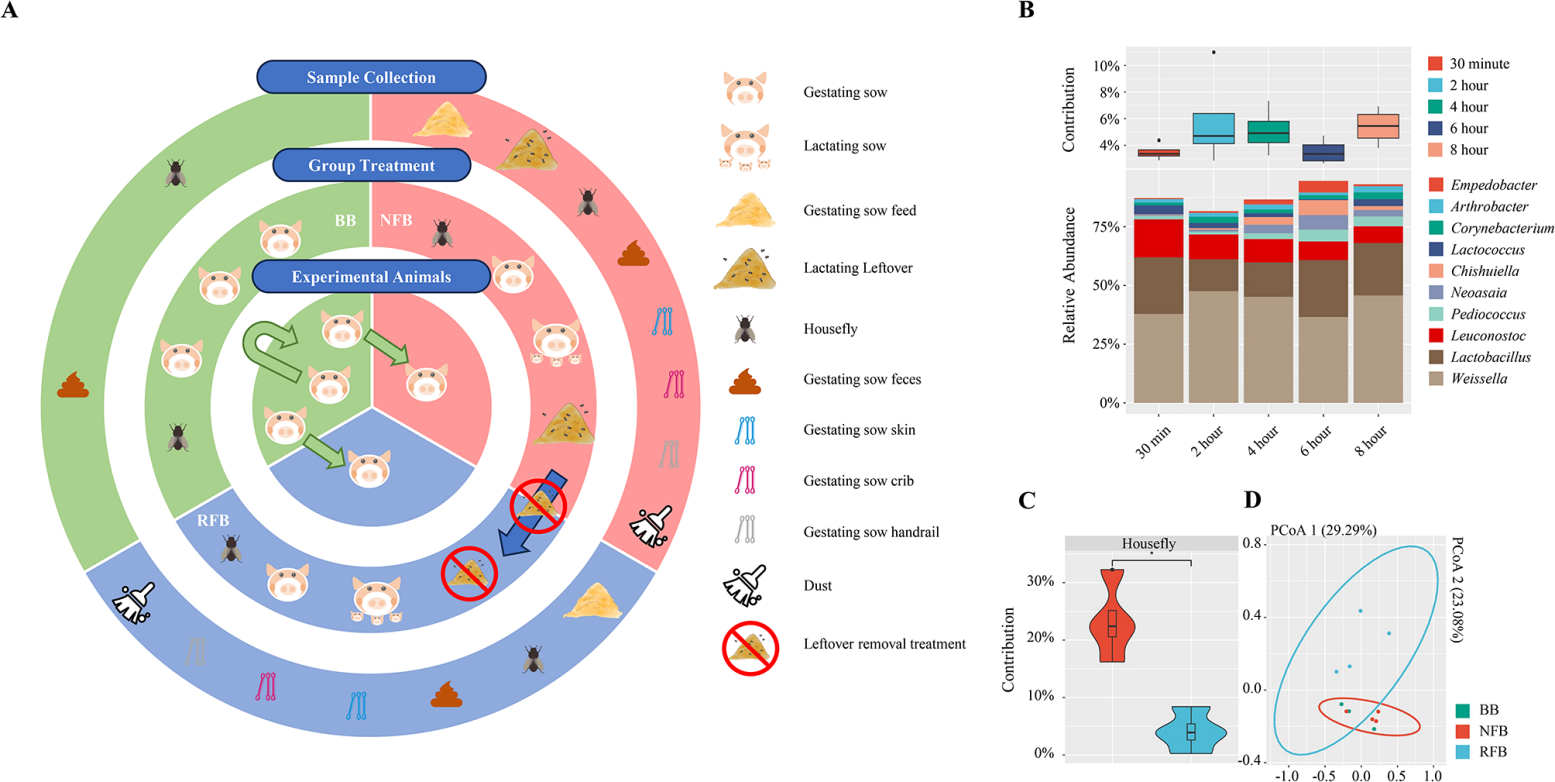
Research design, sample collection and relationship between lactating sow leftover and housefly-carried microbiota. (**A**) Proposed study design. 20 gestating sows from the BB were assigned to BB and NFB when they were 31 days before the FDD, whose feces were collected when they were 21 days before the farrowing due date. After sample collection in the NFB, a 30-day removal lactating sow leftover treatment was implemented in the NFB, which change the farrowing house from NFB to RFB. 10 gestating sows from the BB, which were assign to the RFB at 31 days before the FDD, had feces collected at 21 days before the FDD. In addition, housefly samples were collected in the BB, while housefly, gestating sow feed, lactating sow leftover, environmental microorganisms (gestating sow skin, crib and handrail) and dust were both collected in the NFB and RFB. (**B**) Changes in relative abundance of the top 10 dominant bacteria within the residual feed of lactating sows at the genus level and microbial contribution to housefly-carried microbiota at different time spots. (**C**) Removal of lactating sow leftover significantly reduced the contribution of residual microorganisms to housefly-carried microbiota. (**D**) Principal coordinate analysis of housefly-carried microbiota among BB, NFB and RFB. BB, breeding barn; NFB, non-removal of leftover feed farrowing barn; RFB, removal of leftover feed farrowing barn; FDD, farrowing due date

The present experiment was conducted in a medium-sized swine farm in Jinan city, Shandong province, China, protected from populated areas and upon an antibiotic-free policy. The experiment was carried out in summer, when the average daytime temperature exceeded 30℃. We chose the breeding barn and farrowing barn as the experimental areas for this research, where only gestating sows were present in the breeding barn (BB) and both gestating and lactating sows were present in the farrowing barn (Fig. 1A). At the beginning of the experiment, we randomly selected 30 primiparous crossbred gestating sows of the same breed: 20 gestating sows more than 31 days and 10 gestating sows more than 61 days before the farrowing due date (FDD) in the BB. All experimental gestating sows (inseminated at body weight = 125 kg ± 2.5 kg) lived in the BB from insemination to 31 days before the FDD. At 31 days before FDD, 10 of the first 20 gestating sows were retained in the BB and the remaining 10 gestating sows were transferred to the farrowing barn and randomly assigned to individual farrowing beds which were not adjacent to lactating sows to avoid splashing of residual feed from lactating sows into the surrounding regions of gestating sows. Once the collection of microbiological samples was completed, artificial residual removal treatment was performed in the farrowing barn via removing all leftover feed in the cribs of the lactating sows immediately after feeding. The process of daily cleaning up the leftover feed in the farrowing barn lasted for 30 days. During this period the last 10 gestating sows in the breeding barn were transferred to the farrowing barn and randomly assigned to farrowing beds not adjacent to lactating sows when they were 31 days before FDD. The farrowing beds in the farrowing barn were flushed with water before arranging all the gestating sows and the residual feed of previous sows in the crib was removed. All experimental gestating sows were continued to be managed until farrowing, in which experimental animals whose true date of farrowing was more than 2 days apart from the FDD or those with disease or antibiotic use during gestation were excluded, and 25 gestating sows from a total of 30 gestating sows were consequently selected as the target of this research. Among them, 7 gestating sows were transferred to non-removal lactating residual farrowing barn (NFB) at 31 days before the FDD, 8 gestating sows were transferred to residual removal farrowing barn (RFB) at 31 days before the FDD and 10 gestating sows were retained in the BB.

All sows in the farm were fed twice daily (6 a.m. and 3 p.m.) and gestating sows were fed with the restricted feeding strategy, whereas lactating sows were fed with the ad libitum strategy. Gestating sows received BestHuiKe 1405 commercial gestating sow feed mixed with water whereas lactating sows received BestHuiKe 1406 commercial lactating sow feed mixed with water both from Best Influx Science Bioengineering Technology Co. Ltd (Beijing, China). Drinking water for both gestating and lactating sows was obtained from a local municipal water treatment facility and available ad libitum.

### Sample collection

All fecal samples from gestating sows were collected on day 21 before FDD (*n* = 10 in BB, *n* = 7 in NFB, *n* = 8 in RFB). Fecal samples were collected using a sterile knife to cut off the exterior of the feces before sampling the interior to avoid environmental microbial influences. Prior to leftover feed removal treatment, the residual feed at the surface of the lactating sow crib was randomly collected in the farrowing barn at five time points after feeding, including 8 samples after 30 min, 8 samples after 2 h, 8 samples after 4 h, 6 samples after 6 h and 6 samples after 8 h. One sample of gestating sow feed before feeding and two samples of dust from the air circulation equipment were collected in both NFB and RFB (Fig. 1A).

Fly-capturers (with sterile chocolate syrup as attractants) were placed to capture live houseflies, which were immediately placed at -20 ℃. Over 30 houseflies were collected in the BB and over 50 houseflies were collected respectively in the farrowing barn before and after feed leftover removal. We screened the houseflies based on carapace integrity, divided every 10 houseflies into one sample (*n* = 3 in BB, *n* = 4 in NFB, *n* = 4 in RFB). In addition, we sampled environmental microorganisms using sterile cotton swabs dipped in saline solution on the sow accessible organic and inorganic regions of six randomly selected gestating sows in the farrowing barn before and after removal of the residue, including the skin of gestating sows (*n* = 6 in NFB, *n* = 6 in RFB) about ten-centimeter diameter circle on the neck, gestating sow cribs (*n* = 6 in NFB, *n* = 6 in RFB) and gestating sow handrails (*n* = 6 in NFB, *n* = 6 in RFB). All samples were immediately frozen in liquid nitrogen until further use.

### 16 S rRNA sequencing

All samples bacterial DNA was extracted using the Magnetic Soil and Stool DNA Kit (TIANGEN Biotech, Beijing, China) according to manufacturer’s instructions, and obtained DNA samples were stored at -80 °C until further analysis. Sequencing was performed at Allwegene Technology Co. Ltd. (Beijing, China) based on the amplification of the hypervariable V3-V4 region of the *16 S rRNA* bacterial gene using the 338 F/806R barcode primer pair (F: ACTCCTACGGGAGGCAGCAG; R: GGACTACHVGGGTWTCTAAT). Amplified libraries were sequenced using an Illumina MiSeq PE300 platform (Illumina, San Diego, USA) by Allwegene Co., Ltd.

### Statistical analysis

Raw paired-end reads were assigned to samples based on their unique barcode, which were subsequently removed together with primer adapters, and the obtained data were analyzed by QIIME2 (v.2021.2) [20]. Data were analyzed with QIIME2 DADA2 plugin for quality trimming, denoising, merging and chimera detection. Amplicon sequence variants (ASVs) were taxonomically classified against DADA2-formatted FASTA files derived from the SILVA database [21] using a similarity cutoff value of 99%. All features related to mitochondria or chloroplasts in taxonomic annotation were removed.

Statistical differences of alpha-diversity metrics, including Shannon and observed species indices, were analyzed by the Wilcoxon rank sum test using R (v.4.0.3). Beta-diversity metrics were calculated by unweighted UniFrac distances, and data visualization was conducted by principal coordinate analysis (PCoA) using R (v.4.0.3). Statistical significance of unweighted UniFrac distances were analyzed by analysis of similarities (ANOSIM) by QIIME2. Linear discriminant analysis coupled with effect size (LefSe) [22] (LDA score > 2) was conducted to analyze the differences between genera and ASVs between groups.

SourceTracker algorithm in R (v.1.0) was used to analyze the source of target microorganisms and evaluate the contribution of a set of environment sources via setting microbial contributors as sources and target receptor as sink [23]. Here, sow residual feed in the NFB at different time points, as well as houseflies in the BB were considered as sources, whereas flies in the NFB and RFB were respectively considered as sink to assess the contribution of the microbiota in lactating sow leftover to housefly-carried microbiota. Environmental samples (including gestating sow skin, crib and handrail) in the RFB, as well as dust and houseflies in the NFB were considered as sources, whereas environmental samples in the NFB were considered as sink to assess the contribution of housefly-carried microbiota to the microbiota found in environmental regions in the NFB. Environmental samples (including gestating sow skin, crib and handrail) in the NFB, as well as dust and houseflies in the RFB were considered as sources, whereas environmental samples in the RFB were considered as sink to assess the contribution of housefly-carried microbiota to the microbiota found in environmental regions in the RFB. The fecal microbiota of gestating sows in the BB and the environmental samples (including gestating sow skin, crib and handrail), dust and gestating sow feed in the NFR were considered as sources to assess the microbial contribution to gestating sow feces as sink in the NFR, while the fecal microbiota of gestating sows in the BB and the environmental samples (including gestating sow skin, crib and handrail), dust and gestating sow feed in the RFB were considered as sources to assess the microbial contribution to gestating sow feces as sink in the RFB. The significance of differences in the microbial contribution and relative abundance of target bacteria between different sample groups was evaluated by Kruskal-Wallis rank sum test with R (v.4.0.3).

## Result

### Relationship between leftover feed in lactating sow cribs and housefly-carried microbiota

Lactating leftover feed at NFB without residual removal treatment was exposed to microbes from the environment and experienced more than 9 h of spontaneous fermentation. The alpha diversity analysis showed that the Shannon index experienced volatility from 30 min to 8 h after the end of feeding, while observed OTU continued to rise in number, but none of the adjacent time spots showed significant differences (Supplementary Table 1). LefSe analysis based on genus level demonstrated dominant bacteria at 30-minute, 2-hour and 4-hour of lactating leftover respectively, suggesting that the microbial composition of residual feed for lactating sows changed over time (Supplementary Fig. 1). Meanwhile, the top ten relative abundance features at genus level in the residual feed on average include *Weissella* (∼ 42.73%), *Lactobacillus* (∼ 19.40%), *Leuconostoc* (∼ 10.65%), *Pediococcus* (∼ 2.66%), *Neoasaia* (∼ 2.59%), *Chishuiella* (∼ 2.28%), *Lactococcus* (∼ 2.27%), *Corynebacterium* (∼ 1.95%), *Arthrobacter* (∼ 1.81%) and *Empedobacter* (∼ 1.70%) (Fig. 1B).

Considering the non-negligible bidirectional microbial transfer between houseflies and lactating sow residual feed, we hypothesized that microorganisms carried by houseflies present in the NFB are associated with lactating sow residual feed. Therefore, we evaluated the contribution of lactating sow residual feed to microorganisms carried by houseflies with SourceTracker. The results showed that the lactating sow residual feed provided ∼ 23.32% of microbiota to houseflies located in the NFB, while the removal of lactating sow residual feed significantly reduced the contribution to houseflies from the lactating sow feed (*p* = 0.021). (Fig. 1C). Meanwhile, the average microbial contribution of the lactating sow residual feed fluctuated over time, with the lowest contribution (∼ 3.49%) at the 30-minute time spot and the highest contribution (5.81%) at the 2-hour time spot (Fig. 1B). We used PCoA and ANOSIM with unweighted UniFrac distance to compare the differences in microbial structure carried by houseflies located in NFB and RFB (Fig. 1D). ANOSIM analysis showed that the presence of lactating sow residual feed influenced the microbial structure of houseflies, but did not yet reach a statistically significant difference (*R* = 0.031, *p* = 0.053).

### Implication of houseflies in the change of farrowing barn environmental microbiota

**Fig. 2:**
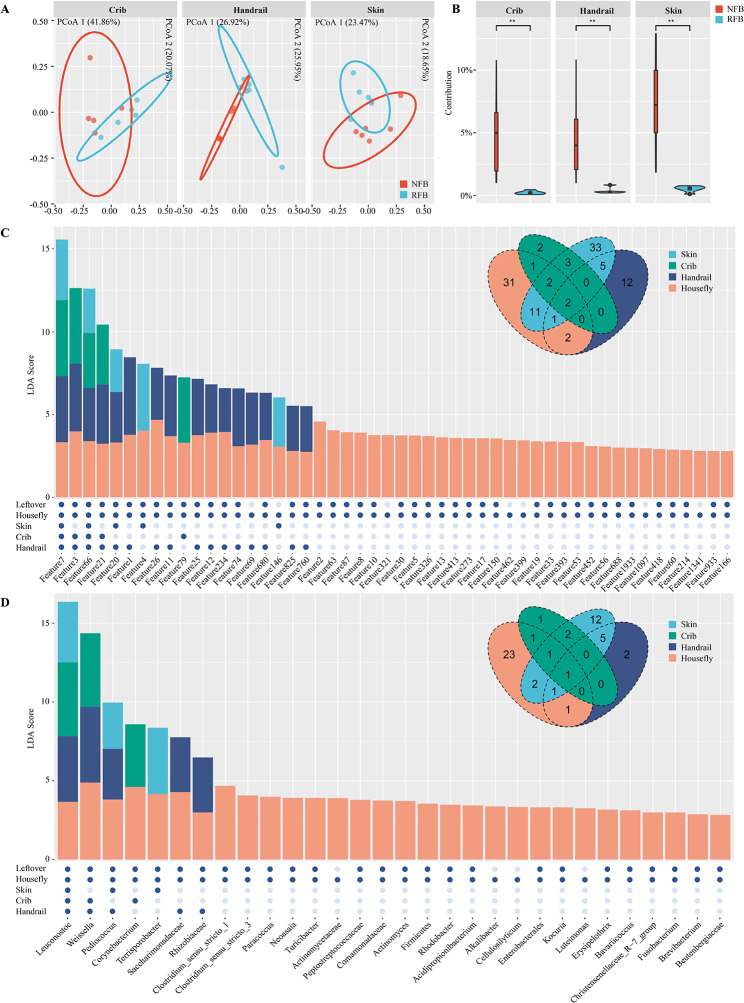
Impact of microbial delivery mediated by houseflies on environmental microbial structure. (**A**) Principal coordinate analysis of microbial environment samples including gestating sow crib, handrail and skin between NFB and RFB. (**B**) Comparison of the contribution of housefly-carried microbiota to environmental microorganisms before and after lactating leftover removal treatment. (**C**) Summation plot of linear discriminant analysis values of dominant bacteria carried by houseflies and dominant bacteria in the environmental region in NFB compared to RFB and Venn plot of common bacteria at the ASV level. (**D**) Summation plot of linear discriminant analysis values of dominant bacteria carried by houseflies and dominant bacteria in the environmental region in NFB compared to RFB and Venn plot of common bacteria at the genus level. BB, breeding barn; NFB, non-removal of leftover feed farrowing barn; RFB, removal of leftover feed farrowing barn; ASV, amplicon sequence variant

We compared microbial component in the gestating sow accessible environmental regions including gestating cribs, handrail and skin between NFB and RFB (Fig. 2A). ANOSIM analyses showed that removal of leftover feed in lactating sow cribs caused significant differences on gestating sow crib surface microbiota (*R* = 0.332, *p* = 0.047), gestating sow handrail surface microbiota (*R* = 0.435, *p* = 0.009), as well as gestating sow skin microbiota (*R* = 0.267, *p* = 0.004). SourceTracker analysis showed that houseflies located in the NFB contributed ∼ 4.97% of microbiota to gestating sow cribs, ∼ 4.67% of microbiota to handrail and ∼ 7.38% of microbiota to gestating sow skin on average, while removal of lactating leftover significantly reduced all the contribution (*p* = 0.004) (Fig. 2B).

We performed LefSe analyses at both ASV and genus level of housefly as well as gestating sow crib, handrail and skin microbiota located at the NFB with their counterparts sampled at the RFB to screen for common dominant features between houseflies and different environmental regions with the presence of leftover feed for lactating sows. At the ASV level, there were 50 dominant target features present in houseflies located at the NFB, of which 43 were found to be present in the lactating sow residual feed. 17 of 43 features were dominant in at least one environmental region located in the NFB and 5 features were dominant in at least two environmental regions located in the NFB, including Feature 7 (*Leuconostoc citreum*), Feature 3 (*Weissella paramesenteroides*), Feature 66 (*Lactobacillus amylotrophicus*), Feature 21 (*Leuconostoc*) and Feature20 (*Pediococcus*). Among them, Feature 7 (*Leuconostoc citreum*) was dominant in the three environmental regions of NFB (Fig. 2C). At the genus level, there were 30 dominant genera present in houseflies located at the NFB, of which 26 were found to be present in the lactating sow residual feed. 7 of 26 genera were dominant in at least one environmental region located in the NFB, and 3 genera were dominant in at least two environmental regions located in the NFB, including *Leuconostoc*, *Weissella* and *Pediococcus*. Among them, *Leuconostoc* was dominant in all three environmental regions of NFB (Fig. 2D).

### Fecal microbiota of gestating sow in different groups

**Fig. 3:**
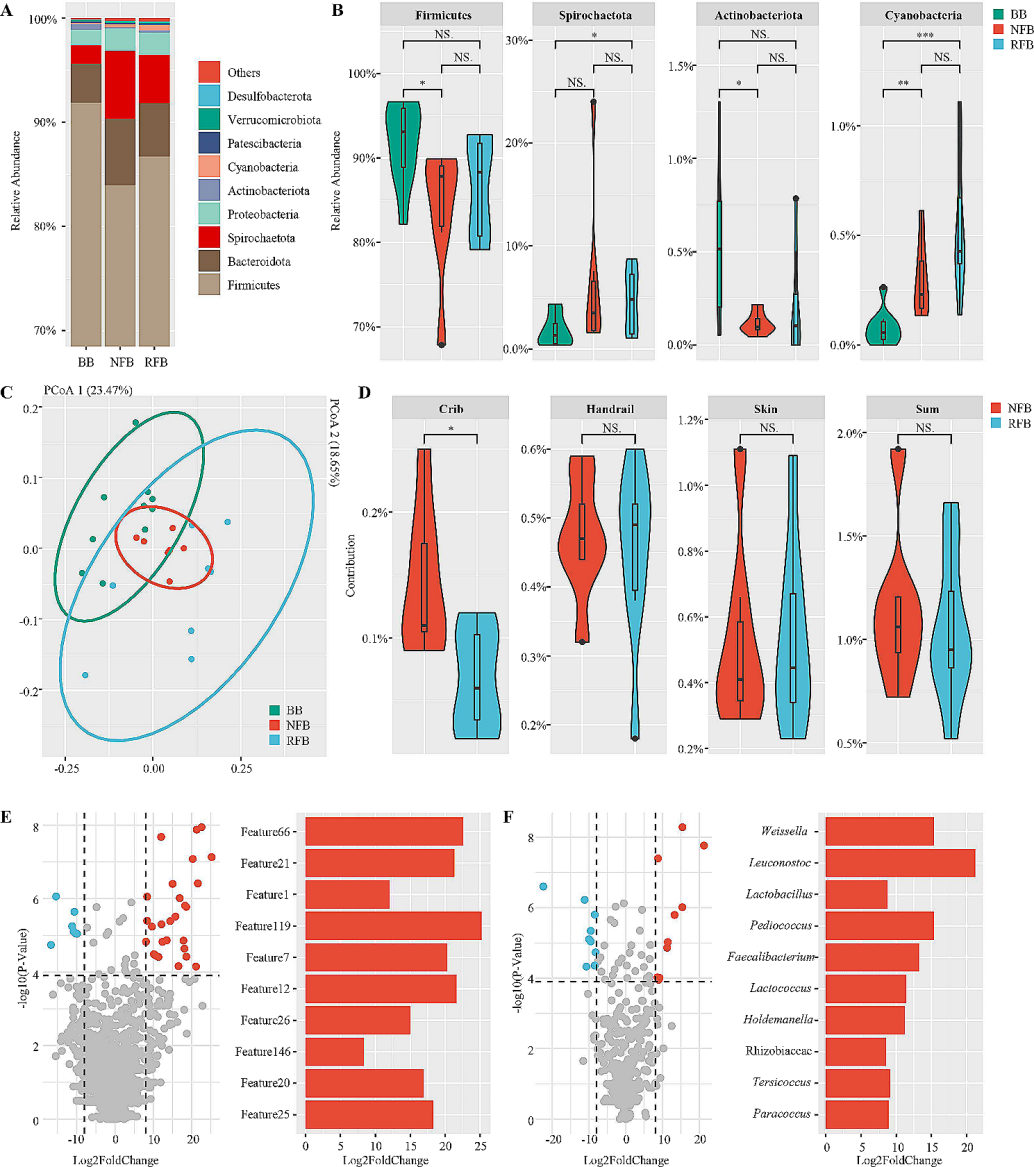
Environmental microbiota modified the microbial structure of fecal bacteria in gestating sows, as well as the effect of residual feed from lactating sows on the global microbiota of the farrowing barn. (**A** and **B**) Differences in the percentage of the top 10 bacteria in relative abundance of fecal bacteria from lactating sows at the phylum level among BB, NFB and RFB. (**C**) Principal coordinate analysis of gestating sow fecal microbiota among BB, NFB and RFB. (**D**) Comparison of NFB and RFB regarding the contribution of different environmental regions including gestating sow crib, handrail and skin to fecal microorganisms in gestating sows. (**E**) Volcano plots of evaluation about the global microbial differences in samples of housefly, gestating sow feces and environmental regions including gestating sow crib, handrail and skin at the ASV level between NFB and RFB. (**F**) Volcano plots of evaluation about the global microbial differences in samples of housefly, gestating sow feces and environmental regions including gestating sow crib, handrail and skin at the genus level between NFB and RFB. BB, breeding barn; NFB, non-removal of leftover feed farrowing barn; RFB, removal of leftover feed farrowing barn; ASV, amplicon sequence variant

Sow fecal microbiota in three groups was dominated by Firmicutes (∼ 87.51%), followed by Bacteroidota (∼ 5.07%), Spirochaetota (∼ 4.31%), Proteobacteria (∼ 1.92%) and Cyanobacteria (∼ 0.30%) in descending order by average relative abundance at the phylum level (Fig. 3A). Firmicutes (*p* = 0.025) and Actinobacteriota (*p* = 0.015) were significantly lower and Cyanobacteria (*p* = 0.003) were significantly higher in the feces of gestating sows located in NFB compared to those located in BB, whereas in the feces of gestating sows located in RFB Spirochaetota (*p* = 0.041) and Cyanobacteria (*p* = 0.001) were significantly increased (Fig. 3B). At the phylum level, there was no significant difference in the relative abundance of fecal microbiota between gestating sows located in NFB and RFB.

Analysis of alpha diversity showed that fecal microorganisms of gestating sows located in both NFB (*p* = 0.008) and RFB (*p* = 0.016) were significantly higher in Shannon’s index than those located in BB (Supplementary Table 2). Meanwhile, fecal microbiota of gestating sows located in NFB and RFB had higher number of observed OTU compared to gestating sows located in BB on average, but only gestating sows located in NFB were significantly higher (*p* = 0.040). In addition, there was no significant difference in alpha diversity between fecal microbiota of gestating sows located at NFB and RFB (Supplementary Table 2).

PCoA and ANOSIM based on unweighted UniFrac distance showed significant differences in fecal microorganisms in all three groups of gestating sows in both comparisons (BB versus NFB, *p* = 0.018; NFB versus RFB, *p* = 0.013; BB vs. RFB, *p* = 0.001) (Fig. 3C). LefSe analysis based on genus level showed that gestating sows located in NFB with the presence of lactating sow leftovers have significant higher *Weissella* (NFB versus BB, *p* = 0.009; NFB versus RFB, *p* = 0.017) and *RF39* (NFB versus BB, *p* = 0.011; NFB versus RFB, *p* = 0.028) in fecal bacteria compared to gestating sows located in BB as well as RFB after removal of residuals, while gestating sows in RFB and BS both upregulated the proportion of *Clostridium sensu stricto 3* (BB versus NFB, *p* = 0.032; RFB versus NFB, *p* = 0.003) and *Anaerocolumna* (BB versus NFB, *p* = 0.034; RFB versus NFB, *p* = 0.016) (Supplementary Figs. 2,3). LefSe analysis based on ASV levels provided analogous results (Supplementary Figs. 4, 5), with removal of residual treatments causing significant decreases in 15 features including Feature 1 (*Weissella*, NFB versus BB, *p* = 0.009; NFB versus RFB, *p* = 0.017) in gestating sow feces, 12 of which were consistent with feces LefSe results of BB versus NFB, and restored 4 features relative abundance in the RFB which are also dominate in the BB comparing to NFB (Supplementary Table 3).

SourceTracker analysis showed that gestating sow cribs, handrails and skins at the NFB collectively provided ∼ 1.14% of microbiota for gestating sow fecal bacteria comparing to ∼ 1.05% at the RFB on average, which did not result in a significant difference (Fig. 3D). Comparing the different environmental regions separately, the contribution of microorganisms was significantly higher in the crib in the NFB than in the RFB (*p* = 0.023), whereas the remaining environmental regions did not show significant changes before and after removal of lactating sow residuals (Fig. 3D).

### Microbial transmission derived from lactating sow leftover

We evaluated each bacterial microorganism as individual microbial transmission pathway along different kinds of sample targets (housefly, gestating sow crib, handrail, skin and gestating sow feces) at the NFB and RFB and used an exponential function summation to accrue differences in relative abundance. We used 3.903 as a probability screening threshold (exponential value of 0.05 for the equivalent 3 groups) and 8 as a multiplicative difference screening threshold (mean 2-fold for the equivalent 3 groups) for screening. At the ASV level, we screened 27 eligible dominant features located in the NFB from 5852 features, of which half of 10 samples with the lowest probability index were top 10 relative abundance features in the residual feed of lactating sows including Feature 21 (*Leuconostoc*), Feature 1 (*Weissella*), Feature 7 (*Leuconostoc citreum*), Feature 12 (*Lactobacillus brevis*) and Feature 20 (*Pediococcus*) (Fig. 3E). At the genus level, we screened 10 eligible dominant genera located in the NFB from 627 genera, half of 10 genera were also the top 10 genera in terms of relative abundance in lactating sow residual feed, including *Weissella*, *Leuconostoc*, *Lactobacillus*, *Pediococcus* and *Lactococcus* (Fig. 3F). We analyzed the correlation between the transmission possibility of target bacteria exceeding the threshold and their relative abundance in the residual feed of lactating sows. Spearman correlation coefficients showed that the correlation between exponential summation of the genus level target bacteria probability index and their residual abundance increased over time, with a coefficient of 0.750 for 30-minute and 2-hour time spots and the coefficient increased to 0.770 at 4 and 6-hour as well as at 8-hour time spots. Based on the level of the ASV correlation analysis suggested that the highest correlation coefficient of 0.370 was found at 8-hour time spot.

To validate the hypothesized microbial transmission chain, we screened for the bacteria present in the lactating sow residual feed whose average relative abundance share synchronous decline in at least one environmental region (gestating sow crib, handrail or skin) and in both housefly and lactating sow feces in the RFB comparing to the NFB. We hypothesized that these bacteria come from the leftover of lactating sows and spread to different farrowing barn environmental regions with the help of houseflies, ultimately affecting the fecal microbiota of gestating sows. At the ASV level, Feature 1 (*Weissella*), Feature 4 (*Terrisporobacter*), Feature 5 (Peptostreptococcaceae), Feature 25 (*Streptococcus*), Feature 39 (*Weissella*) and Feature 40 (*Terrisporobacter*) shared synchronous decline both in housefly-skin-sow feces and housefly-handrail-sow feces samples. Target features in housefly-crib-sow samples were similar except that feature 40 was replaced by feature 22 (*Escherichia-Shigella*) (Supplementary Table 4). At the genus level, nine genera from lactating leftover including *Weissella*, *Streptococcus*, *RF39*, *Cellulosilyticum*, *UCG-002*, *UCG-005*, *Terrisporobacter*, *Escherichia-Shigella* and Peptostreptococcaceae are synchronized with a decline in housefly-skin-sow feces samples of RFB. In housefly-crib-sow feces and housefly-handrail-sow feces samples, the number of eligible target genera dropped to four and five respectively. Among them, *Weissella* showed a significant decrease in all kinds of sample (Supplementary Table 5).

## Discussion

The 30-day-long lactating leftover removal treatment significantly changed the environmental microbial structure in different regions of the farrowing barn, suggesting the leftover is an important contributor to the environmental microbiota in the farrowing barn. Our environmental samples were collected in regions away from the lactating sows’ farrowing beds, as well as the sows were fed with wet feed which avoid microbial transmission caused by splashing or dusting of residual feed from cribs. Therefore, the effect of leftover feed on the microbial structure of the farrowing barn environment should be dependent on the microbial transmission medium.

The association between leftover and environmental microbial structure was evidenced with the results of microbial abundance and difference probability (Fig. 3E and F). Half of the top ten bacteria with differential cumulative probability involving the housefly, environment and sow samples of NFB, both at the genus level and ASV level, were dominant in lactating residual feed (top 10 in relative abundance). Meanwhile, Spearman’s correlation analysis demonstrated the correlation between the differential cumulative probability of these eligible dominant bacteria and their relative abundance in the residual feed increased over time and reached the highest value at the 8-hour time point, suggesting that there should be a sustained and cumulative effect of the residual feed on the environmental microbiota.

Houseflies are ubiquitous and synanthropic, which allows them to transport and disperse microorganisms in urban or natural environments [24]. The removal treatment of leftover, for one thing, blocks the physical contact between the adult houseflies and leftover microbes during the feeding process. Since houseflies have the habit of laying their eggs within the food [25], the microorganisms in the food can colonize the gut during the larval stage of the housefly [26]. Therefore, the removal of the residues, for another, results in newborn houseflies’ unavailability of microbes in the lactating sow residual feed in the RFB during their lifecycle, which collectively cause microbial difference between houseflies in the NFB and RFB. Paradoxically, the structural differences in microorganisms of houseflies before and after leftover removal treatments were smaller than the structural differences in microorganisms in individual environmental regions in the farrowing barn, as a transmitter of microorganisms. Previous studies have shown that the microbial structure of microbes in the body of houseflies (including in the digestive tract) is more stable than that of their surface microbes [27], while certain microbes can maintain for more than 30 days [28]. Therefore, performing a 30-day removal residue treatment may have altered the microbes mainly on the body surface of houseflies, resulting in differences in microbial transmission, but the effect of internal microbes was relatively low. In this research, we use 16 S rRNA gene amplicon sequencing of housefly-carried bacteria by whole pulverization of houseflies, which means the microbiota on the surface and the internal compartments of houseflies was both evaluated. On the other hand, the expected life span of houseflies is more than 30 days [29], making the houseflies we collected in RFB after residual removal treatment were likely to be exposed to lactating sow residual feed during their life cycle. The above factors may explain why the differences in microbial structure of houseflies were not as expected.

Previous studies have widely documented the role of houseflies in the transmission of pathogenic bacteria such as *Staphylococcus* [30] and *Streptococcus* [31]. In addition, houseflies in farms during summer have been shown to transmit microorganisms within 5–7 km during their life cycle [32]. However, the role of houseflies in the modification of environmental microorganisms under enclosed areas has not been evaluated. With LefSe analysis of houseflies and different environmental regions to find common target microorganisms, the results based on ASV levels showed that houseflies were more adept at spreading 70% dominant bacteria in lactating residual feed (top 10 relative abundance) than traditional pathogenic microorganisms including *Staphylococcus saprophyticus* (Feature 79) and *Streptococcus* (Feature 25). Intriguingly, these dominant bacteria are *Leuconostoc* (Feature 7, 21), *Weissella* (Feature 1, 3), *Pediococcus* (Feature 20) and *Lactobacillus* (Feature 11,12), all belonging to Firmicutes and capable of producing exopolysaccharides [33], suggesting that exopolysaccharides may help certain microorganisms utilize houseflies for environmental transmission. However, after residual removal treatment, these bacteria significantly decreased in the environment as the microbial source was blocked, suggesting the indispensable significance of houseflies in maintaining the relative abundance of these target microorganisms in the farrowing barn.

The absence of the AIAO system resulted in the mixing feeding of gestating and lactating sows in the farrowing house. In this research, we further explored the effects of environmental microbial changes on gestating sows. 15 kinds of bacterial features were significantly reduced in the fecal bacteria of gestating sows after removal treatment, while 12 of these bacteria were also significantly reduced in the fecal bacteria of BB gestating sows where only gestating sows present, demonstrating that the effect of mixing feeding on the gestating sow fecal microbial composition was attenuated by residual removal treatment. However, only *Weissella* (Feature 1) showed a synchronized significant decrease in relative abundance among housefly, environment (handrail) and gestating sows feces samples, which may be dependent on the potent digestive tract colonization ability possessed by *Weissella* [34] and sow self-resistance of digestive tract to environmental microorganisms.

However, the average relative abundance of potentially pathogenic bacteria including *Escherichia-Shigella* and *Streptococcus* were synchronized to increase among houseflies, environment and feces samples of gestating sows with the presence of lactating leftover feed. *Escherichia-Shigella* has been suggested to be a hallmark of intestinal flora dysbiosis [35], while maternal *Streptococcus* colonization can negatively impact on the offspring in the gestation period [36]. In contrast, removal of the residual feed significantly restored the relative abundance of *Christensenellaceae_R-7_group* in gestating sow feces located in RFB, which was closely associated with body health [37]. In conclusion, the leftover removal treatment for lactating sows can alleviate the negative effects due to the absence of the AIAO system in the farrowing barn. Due to current limited experimental conditions, we have not further evaluated the effects of AIAO deficiency on production performance in the farrowing house in this research. We will further explore the effects of changes in the microbial structure of the environment caused by residual feed in the farrowing barn on the health of offspring piglets in the future study and increase the sample capacity.

## Conclusion

Lactating sow leftover is an important source of environmental microorganisms in the farrowing barn, whose transmission is mediated by houseflies. Gestating sow fecal microbiota was modified with the existence of lactating sow leftover due to mixing feeding of gestating and lactating sows in one barn caused by the absence of the AIAO system. Timely removal of leftovers for 30 days can alleviate the effects of lactating sow feed on gestating sows.

### Electronic supplementary material

Below is the link to the electronic supplementary material.

**Supplementary Material 1: Supplementary Table 1.** Alpha diversity of lactating sow leftover microbiota at different time spots

**Supplementary Material 2: Supplementary Table 2.** Alpha diversity of gestating sow fecal microbiota among the breeding barn, non-removal of leftover feed farrowing barn and removal of leftover feed farrowing barn

**Supplementary Material 3: Supplementary Table 3.** Common dominant features of fecal samples in the NFB versus BB and RFB and common dominant features of fecal samples in the BB and RFB versus NFB with linear discriminant analysis coupled with effect size at the amplicon sequence variant level. BB, breeding barn; NFB, non-removal of leftover feed farrowing barn; RFB, removal of leftover feed farrowing barn

**Supplementary Material 4: Supplementary Table 4.** Relative abundance of bacteria in lactating sow leftover whose average relative abundance share synchronous decline in at least one environmental region (gestating sow crib, handrail or skin) and in both housefly and lactating sow feces in the removal of leftover feed farrowing barn compared to the non-removal of leftover feed farrowing barn at the amplicon sequence variant level

**Supplementary Material 5: Supplementary Table 5.** Relative abundance of bacteria in lactating sow leftover whose average relative abundance share synchronous decline in at least one environmental region (gestating sow crib, handrail or skin) and in both housefly and lactating sow feces in the removal of leftover feed farrowing barn compared to the non-removal of leftover feed farrowing barn at the genus level

**Supplementary Material 6: Supplementary Figure 1.** Linear discriminant analysis coupled with effect size of the lactating sow leftover microbiota at different time spots at the genus level

**Supplementary Material 7: Supplementary Figure 2.** Linear discriminant analysis coupled with effect size of the gestating sow fecal microbiota between breeding barn and non-removal of lactating residual farrowing barn at the amplicon sequence variant level

**Supplementary Material 8: Supplementary Figure 3.** Linear discriminant analysis coupled with effect size of the gestating sow fecal microbiota between non-removal of lactating residual farrowing barn and removal of lactating residual farrowing barn at the amplicon sequence variant level

**Supplementary Material 9: Supplementary Figure 4.** Linear discriminant analysis coupled with effect size of the gestating sow fecal microbiota between breeding barn and non-removal of lactating residual farrowing barn at the genus level

**Supplementary Material 10: Supplementary Figure 5.** Linear discriminant analysis coupled with effect size of the gestating sow fecal microbiota between non-removal of lactating residual farrowing barn and removal of lactating residual farrowing barn at the genus level

## Data Availability

The data that support the findings of this study have been deposited on NCBI with accession number PRJNA905728.

## Abbreviations

ASV: Amplicon sequence variant
PCoA: Principal coordinate analysis
ANOSIM: Analysis of similarity
LefSe: Linear discriminant analysis coupled with effect size
BB: Breeding barn
NFB: Non-removal of leftover feed farrowing barn
RFB: Removal of leftover feed farrowing barn
AIAO: All-in/all-out
FDD: Farrowing due date
