# Supplementary figures and images for: Removal of leftover feed shapes environmental microbiota and limits houseflies-mediated dispersion of pathogenic bacteria in sow breeding farms
Source: Anim Microbiome. 2024 Mar 5;6:10. doi: 10.1186/s42523-024-00296-6 (PMC10913660; doi:10.1186/s42523-024-00296-6)

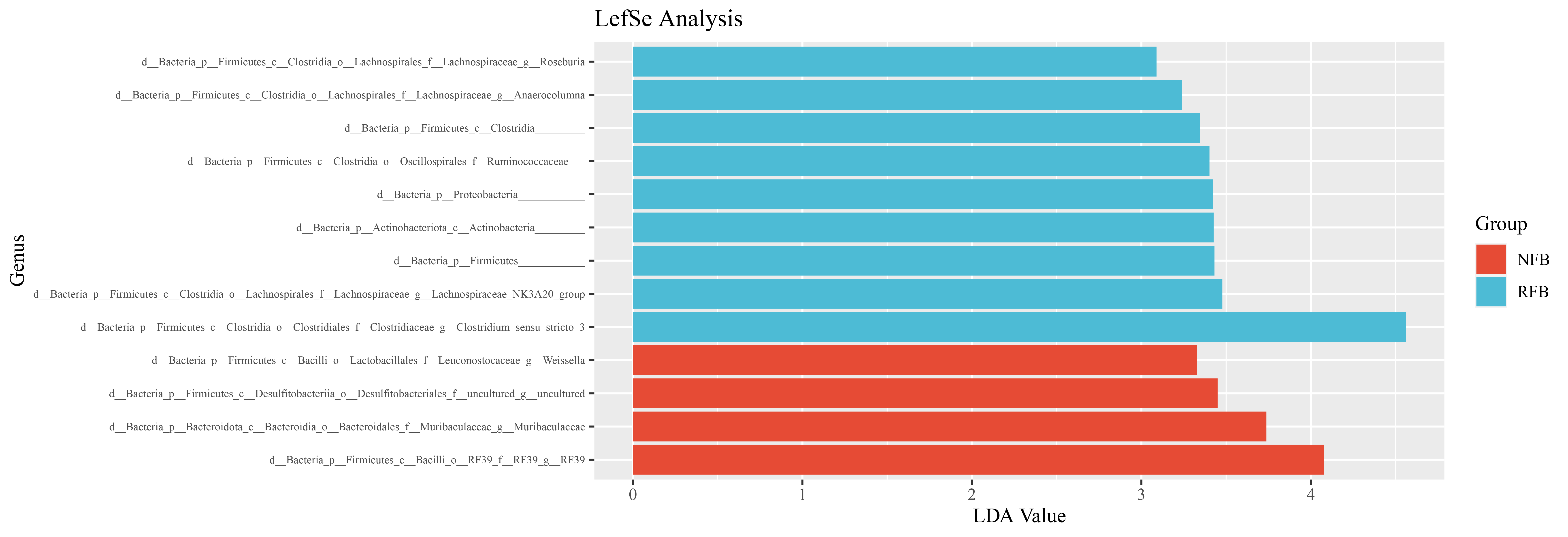

Supplement: Supplementary file 6 — Supplementary Material 6: Supplementary Figure 1. Linear discriminant analysis coupled with effect size of the lactating sow leftover microbiota at different time spots at the genus level [file 42523_2024_296_MOESM6_ESM.png]

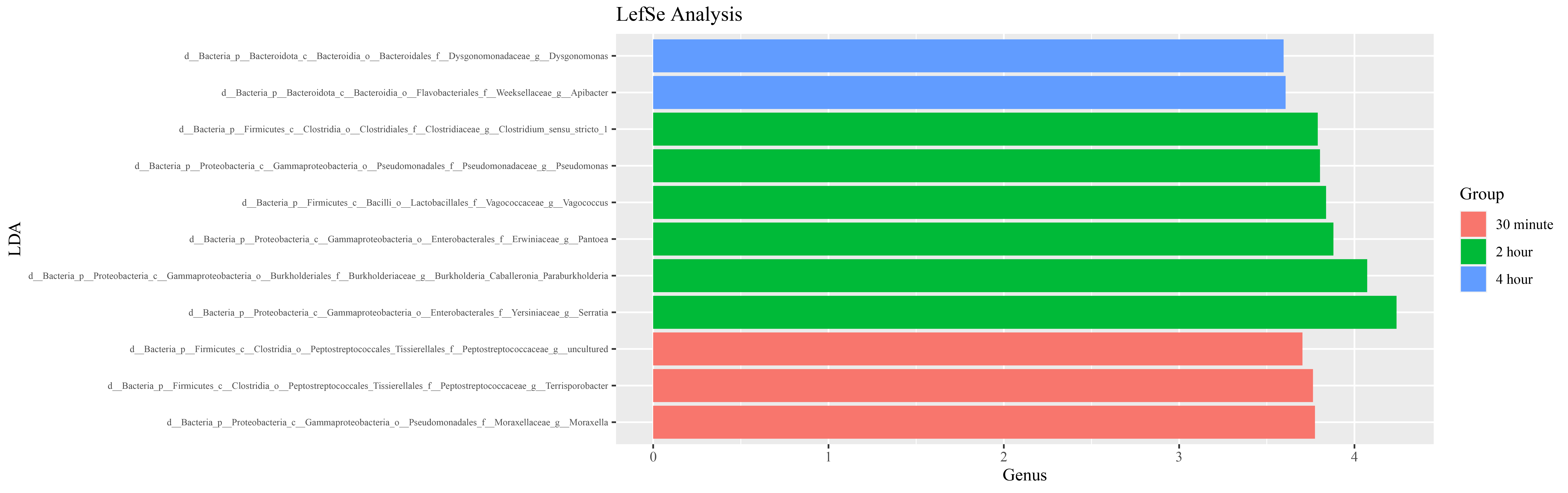

Supplement: Supplementary file 7 — Supplementary Material 7: Supplementary Figure 2. Linear discriminant analysis coupled with effect size of the gestating sow fecal microbiota between breeding barn and non-removal of lactating residual farrowing barn at the amplicon sequence variant level [file 42523_2024_296_MOESM7_ESM.png]

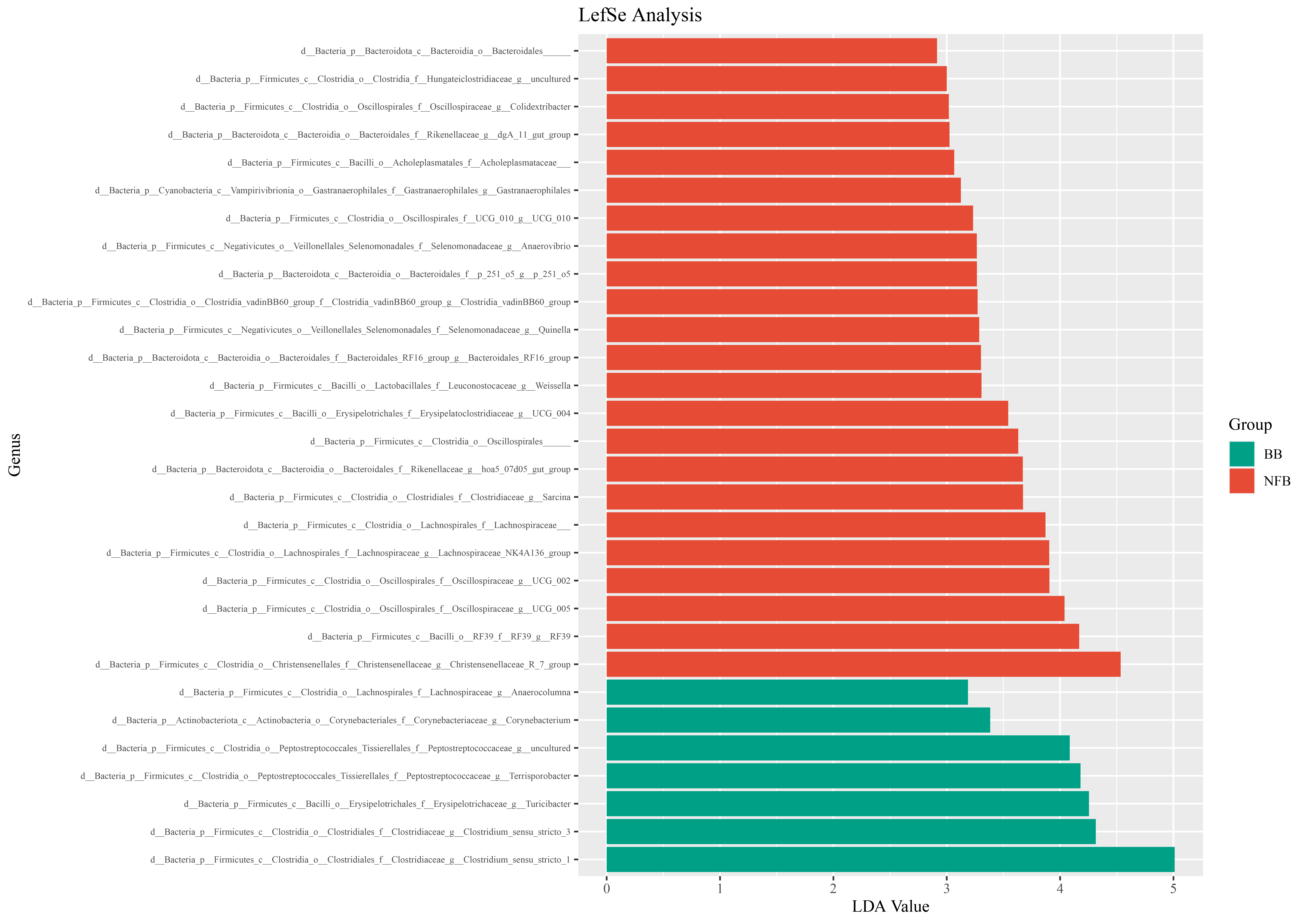

Supplement: Supplementary file 8 — Supplementary Material 8: Supplementary Figure 3. Linear discriminant analysis coupled with effect size of the gestating sow fecal microbiota between non-removal of lactating residual farrowing barn and removal of lactating residual farrowing barn at the amplicon sequence variant level [file 42523_2024_296_MOESM8_ESM.png]

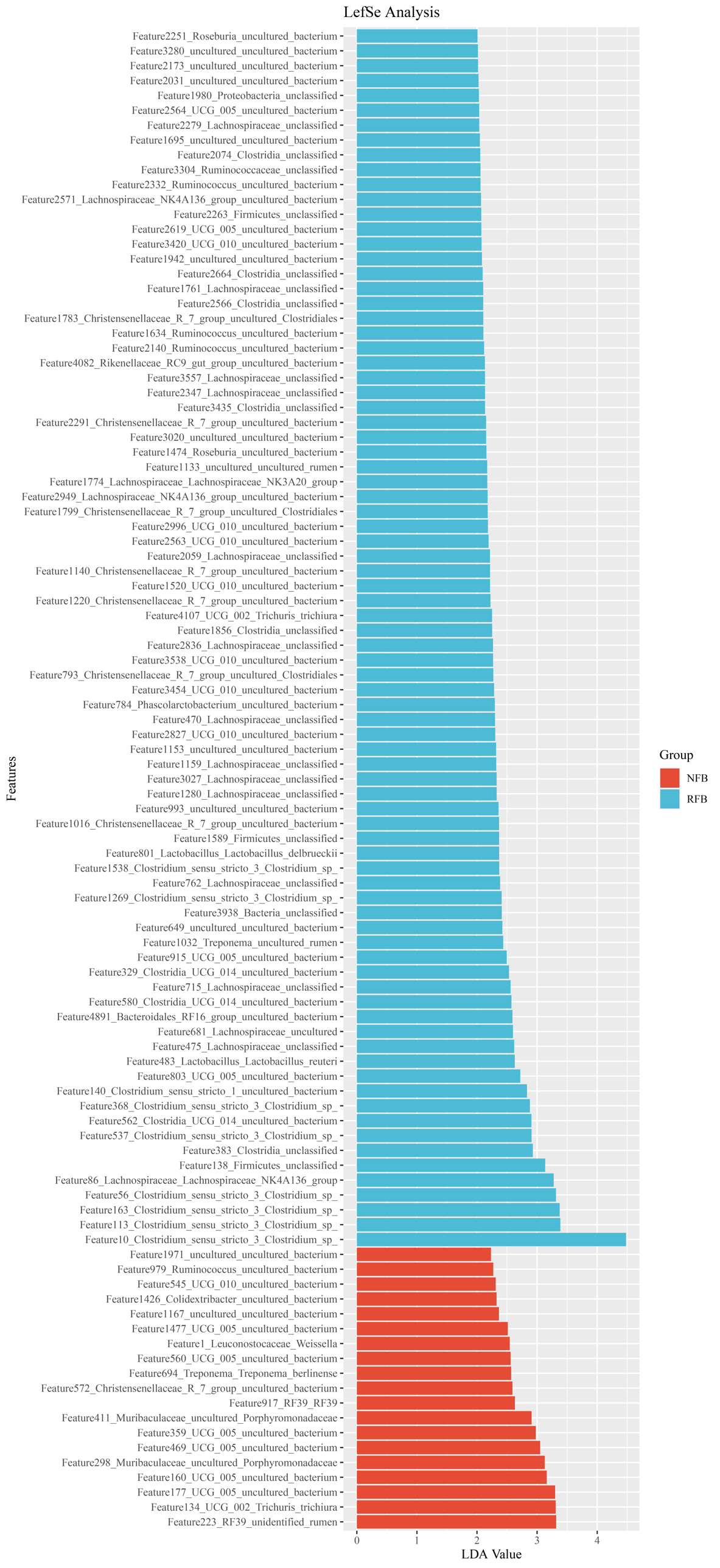

Supplement: Supplementary file 9 — Supplementary Material 9: Supplementary Figure 4. Linear discriminant analysis coupled with effect size of the gestating sow fecal microbiota between breeding barn and non-removal of lactating residual farrowing barn at the genus level [file 42523_2024_296_MOESM9_ESM.png]

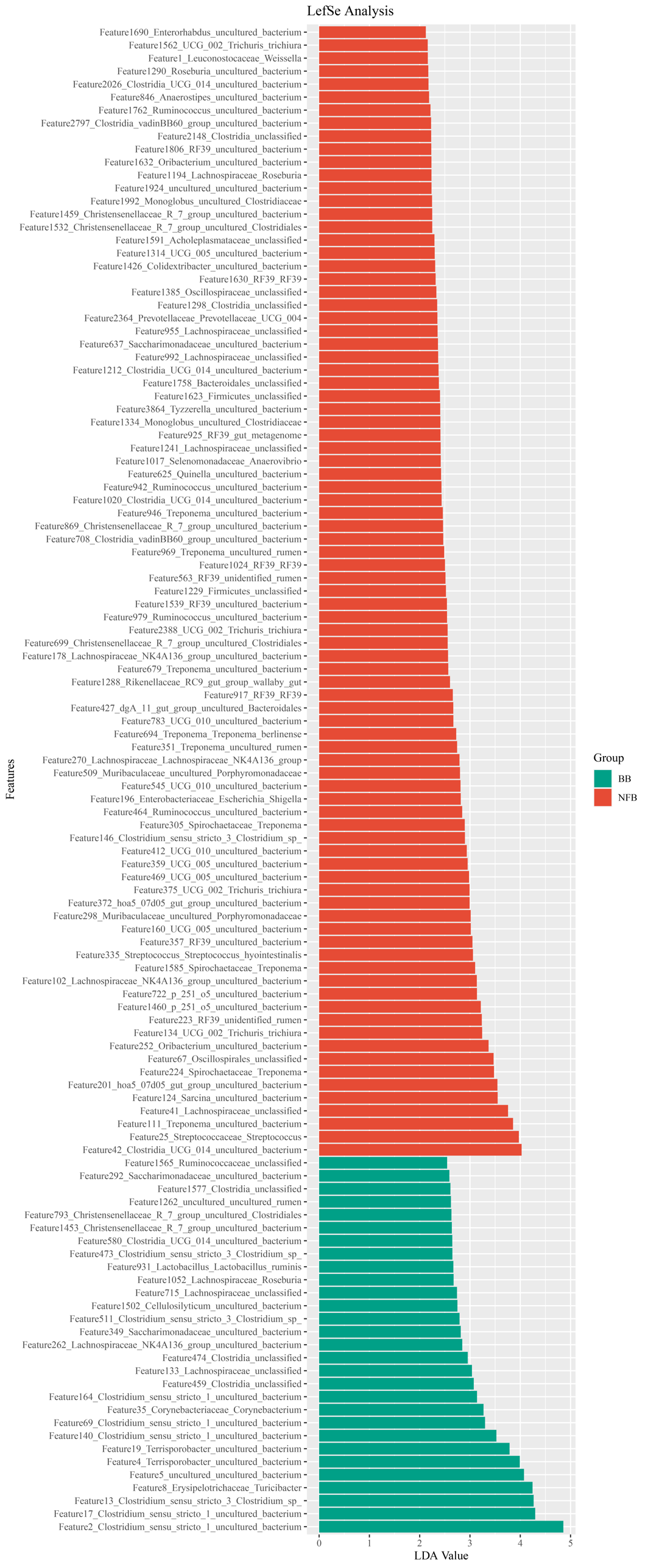

Supplement: Supplementary file 10 — Supplementary Material 10: Supplementary Figure 5. Linear discriminant analysis coupled with effect size of the gestating sow fecal microbiota between non-removal of lactating residual farrowing barn and removal of lactating residual farrowing barn at the genus level [file 42523_2024_296_MOESM10_ESM.png]
